# Supplementary material for: Slow fluctuations in ongoing brain activity decrease in amplitude with ageing yet their impact on task-related evoked responses is dissociable from behavior
Source: eLife. 2022 May 24;11:e75722. doi: 10.7554/eLife.75722 (PMC9129875; doi:10.7554/eLife.75722)
Supplement: Transparent reporting form [file elife-75722-transrepform1.pdf]

Westbrook Centre
Milton Road
Cambridge CB4 1YG, UK

P 01223 855340
W elifesciences.org
T @elife

eLife's transparent reporting form

We encourage authors to provide detailed information *within their submission* to facilitate the interpretation and replication of experiments. Authors can upload supporting documentation to indicate the use of appropriate reporting guidelines for health-related research (see EQUATOR Network), life science research (see the BioSharing Information Resource), or the ARRIVE guidelines for reporting work involving animal research. Where applicable, authors should refer to any relevant reporting standards documents in this form.

If you have any questions, please consult our Journal Policies and/or contact us: editorial@elifesciences.org.

Sample-size estimation

- You should state whether an appropriate sample size was computed when the study was being designed
- You should state the statistical method of sample size computation and any required assumptions
- If no explicit power analysis was used, you should describe how you decided what sample (replicate) size (number) to use

Please outline where this information can be found within the submission (e.g., sections or figure legends), or explain why this information doesn't apply to your submission:

> For this study, we used a dataset acquired for our previous study. Sample size computations were not performed. Based on previous studies investigating the effect of ageing on the variability of evoked responses, we estimated that the available sample size would be appropriate (e.g. Tran et al. 2020 J Cogn Neurosci. Sep;32(9):1813-1822; Sander et al. 2012 Neuroimage. Jan 2;59(1):646-54).

Replicates

- You should report how often each experiment was performed
- You should include a definition of biological versus technical replication
- The data obtained should be provided and sufficient information should be provided to indicate the number of independent biological and/or technical replicates
- If you encountered any outliers, you should describe how these were handled
- Criteria for exclusion/inclusion of data should be clearly stated
- High-throughput sequence data should be uploaded before submission, with a private link for reviewers provided (these are available from both GEO and ArrayExpress)

Please outline where this information can be found within the submission (e.g., sections or figure legends), or explain why this information doesn't apply to your submission:

> Raw EEG and pupil data are available in the OpenNeuro Repository - https://openneuro.org/datasets/ds003690/versions/1.0.0
> Maria J. Ribeiro and Miguel Castelo-Branco (2021). EEG, ECG and pupil data from young and older adults: rest and auditory cued reaction time tasks. OpenNeuro. [Dataset] doi: 10.18112/openneuro.ds003690.v1.0.0
>
> Criteria for exclusion/inclusion of data, including how we handled outliers, are clearly stated in the methods section.

eLife Sciences Publications, Ltd is a limited liability non-profit non-stock corporation incorporated in the State of Delaware, USA, with company number 5030732, and is registered in the UK with company number FC030576 and branch number BR015634 at the address Westbrook Centre, Milton Road, Cambridge CB4 1YG.

Statistical reporting

- Statistical analysis methods should be described and justified
- Raw data should be presented in figures whenever informative to do so (typically when N per group is less than 10)
- For each experiment, you should identify the statistical tests used, exact values of N, definitions of center, methods of multiple test correction, and dispersion and precision measures (e.g., mean, median, SD, SEM, confidence intervals; and, for the major substantive results, a measure of effect size (e.g., Pearson's r, Cohen's d)
- Report exact p-values wherever possible alongside the summary statistics and 95% confidence intervals. These should be reported for all key questions and not only when the p-value is less than 0.05.

Please outline where this information can be found within the submission (e.g., sections or figure legends), or explain why this information doesn't apply to your submission:

For the main analyses, the N, the statistical methods used, and their results are described in the methods and results sections. For supplementary analyses, statistical results are described in the supplementary figure legends.<br>Figures show all data points whenever informative.

(For large datasets, or papers with a very large number of statistical tests, you may upload a single table file with tests, Ns, etc., with reference to sections in the manuscript.)

Group allocation

- Indicate how samples were allocated into experimental groups (in the case of clinical studies, please specify allocation to treatment method); if randomization was used, please also state if restricted randomization was applied
- Indicate if masking was used during group allocation, data collection and/or data analysis

Please outline where this information can be found within the submission (e.g., sections or figure legends), or explain why this information doesn't apply to your submission:

Samples were allocated to experimental groups based on the participant's age. Masking was not used for group allocation or data collection, as it was not possible to mask participants' age. Data analyses were mostly done with computer scripts and therefore the same independent of group allocation. Manual EEG and pupil data cleaning was performed in both groups of participants in parallel, alternating between the two groups as to avoid the introduction of bias resulting from analysing one group first and another after.

Additional data files ("source data")

- We encourage you to upload relevant additional data files, such as numerical data that are represented as a graph in a figure, or as a summary table
- Where provided, these should be in the most useful format, and they can be uploaded as "Source data" files linked to a main figure or table
- Include model definition files including the full list of parameters used
- Include code used for data analysis (e.g., R, MatLab)
- Avoid stating that data files are "available upon request"

Please indicate the figures or tables for which source data files have been provided:

eLife Sciences Publications, Ltd is a limited liability non-profit non-stock corporation incorporated in the State of Delaware, USA, with company number 5030732, and is registered in the UK with company number FC030576 and branch number BR015634 at the address Westbrook Centre, Milton Road, Cambridge CB4 1YG.

The dataset used in this study has been made available in Open Neuro Repository under the doi: 10.18112/openneuro.ds003690.v1.0.0.

All analyses and figures presented in the manuscript resulted from analyses of this dataset.

Matlab code used for data analyses is publicly available on GitHub https://github.com/CIBIT-ICNAS/2021_Ribeiro_BrainVariability_Aging.

eLife Sciences Publications, Ltd is a limited liability non-profit non-stock corporation incorporated in the State of Delaware, USA, with company number 5030732, and is registered in the UK with company number FC030576 and branch number BR015634 at the address Westbrook Centre, Milton Road, Cambridge CB4 1YG.
